# The Interrelation between Orthodontic Malformations and Diseases of the Nose and Throat

**Published:** 1920-02

**Authors:** Geo. C. Dittman

**Affiliations:** St. Paul, Minn.


### THE INTERRELATION BETWEEN ORTHODONTIC MALFORMATIONS AND DISEASES OF THE NOSE AND THROAT

BY GEO. C. DITTMAN, M.D., ST. PAUL, MINN.

That malformations of the dental arches and the maxillae are a great etiologic factor in producing many nose and throat affections may be realized by a review of the closely correlated anatomy of the bones forming the face.

To better understand this orthodontic interrelationship a brief embryologic survey is necessary. The evolution of the face depends largely upon the parts concerned in the formation of the mouth and nose. The first step in the differentiation of the face is the formation of the oral plate which makes its appearance on the twelfth day, later this becomes the mouth. At the third week the first and second visceral arches appear, the first arch divides into the mandibular arch and the maxillary process, the second arch springs from the mandibular arch; as these processes grow toward the median line the maxillary process becomes the upper jaw and the mandibular process the lower jaw. At the second month a groove makes its appearance on the frontal protuberance and on each side of this there is formed two nasal processes, the outer pair becomes the outer wall of the nose, and the inner pair the septum, as these processes grow downward the union of the two upper portions of the mandibular arch forms the floor of the nose.

By the second month of intrauterine life the septum becomes cartilaginous, and by the third month ossification begins in the vomer, into the groove of the vomer the perpendicular plate of the ethmoid fits anteriorly, the rostrum of the sphenoid unites above and the nasal crest of the superior maxillary and palate bones unite below.

At birth the ethmoid part which goes to make up the nasal space is higher than the maxillary portion, but it becomes of equal dimensions with age, this increase is due to the descent of the hard palate. As the teeth erupt the nares develop, the superior maxillae enlarge and the antrum of Highmore forms, gradually increasing in size; with enlargement of the nasal chambers and formation of the alveolar process development is completed.

It will be noted that the septum is the last of the facial bones to ossify, the fact that ossification begins posteriorly explains the rarity of deformities in this part of the bone.

Thus the nasal chamber depends wholly upon the proper growth of these processes and the adjoining parts and any pathologic condition or malformation tends to affect either by extension or anatomical conformation the sinuses or orbital cavities with subsequent effect upon the ears interrelated as they are with these parts.

In the examination of the nares a deflected septum will be noted, but often if the oral vault is examined it will be found highly arched and the alveolar processes close together.

I have often noted that when doing the submucous operation for deflected septum where the maxillary ridge is thickened and broad, there is usually associated with the condition a high-arched vault, and it must be further remarked that these are the most difficult bony ridges to remove, considerable hemorrhage takes place, and when a chisel is used it may be broken in the attempt to level the ridge.

A high-arched vault with narrow alveolar processes must tend to so crowd the turbinate bodies and septum together that the respiratory space within the nose becomes greatly decreased, which in turn causes mouth breathing and its subsequent results—dry pharynx, enlarged tonsils, adenoids and fetid breath.

It is stated that tonsils and adenoids are the most common cause for mouth breathing in the young, but is it not possible, because of anatomic nasal defects that the enlargement of these tissues might be due to an increased hyperemia mainly as a result of the orthodontic anomaly? What effect treatment of the dental arches has upon the nasal spaces and septum can be appreciated by the joint observation of orthodontist and rhinologist of these cases.

Trendelenburg states that a persistent high arch of the hard palate is the cause of a deflected septum. Ballenger concluded that it is due to inco-ordination in the development of the bones of the face. This is a fair conclusion when it is realized that the most important area within the nose, and known as the viscous area, is but one inch in diameter, and any obstruction to this region might cause an infection of the nasal sinuses, and because of their close anatomical relationship would produce an intrusion upon or actual rupture into the orbital cavity, although more often monocular, both eyes may be effected.

Ocular complications are more frequent in chronic than acute sinus infections, perhaps the most misleading ocular complaint resulting from sinus involvement is asthenopia or an inability to use the eyes for near work for any length of time.

As the sinues under normal conditions are designed to contain air, any secretion of whatsoever nature remaining for any length of time is pathologic, and the importance of early and free drainage should be recognized.

The throat is primarily affected by attempting to clear it of the increased secretion and mucus, dropping from the posterior nares.

From a neurologic view directly as a result of nasal obstruction or malformation the trigeminus, that great sensory nerve of the head, with its vast number of distributing branches, may be directly affected through reflex conditions.

How best to treat these conditions. In children the tonsils and adenoids, if enlarged, should be removed at an early date, any malformation of the alveolar arches or teeth should be taken care of by the orthodontist; the proper time is during the development of the bony structures and the teeth, since this period extends over several years the greatest importance should be placed on proper occlusion best obtained and constructed at this stage of life.

It can not be advisable to perform a submucous resection of the septum too early in life, or remove enlarged turbinate bodies; it is surprising how rapidly the turbinate bodies will shrink down to normal after removal of the tonsils and adenoids.

With adults who have gone through life with high-arched palate and contracted dental arches, treatment is still a matter for discussion. L. W. Dean, in the *Journal of the American Medical Association*, November 26, 1910, reports a case in which nasal breathing was impossible, but by widening of the palatal arch the patient became a nasal breather; as regards the narrow chambers with deflected septum, enlarged turbinates or polypi secondary to sinus infection, I believe it has been definitely settled that these conditions should be corrected by operation. Perhaps no operation produces such striking results as the submucous operation for the correction of septal deformities where there has been a narrowed nasal chamber with sinus infection and improper areation of the middle ear, the technic has been universally adopted.

CONCLUSIONS

1. This is an era which must recognize dentistry as an aid to medicine and vice versa.

2. This is a subject which closely associates the orthodontist and rhinologist, and for best results in the young a co-operation of the two specialties is imperative.

3. Nasal and throat operations in conjunction with orthodontic correction often give best results to patient.

4. Orthodontic deformities and respiratory function are correlated.—*International Journal of Orthodontia.*

